# VPS34-mediated autophagosome-lysosome fusion facilitates classical swine fever virus replication

**DOI:** 10.1128/jvi.01641-25

**Published:** 2025-11-25

**Authors:** Yu-hang Li, Yan Cheng, Bing-qian Zhao, Jin-xia Chen, Mei-zhen Li, Lin-han Zhong, Qi Dai, Xiao-qing Bi, Jing Chen, Bin Zhou

**Affiliations:** 1 MOE Joint International Research Laboratory of Animal Health and Food Safety, College of Veterinary Medicine, Nanjing Agricultural University 261674, Nanjing, China; 2 Key Laboratory of Animal Bacteriology, Nanjing Agricultural University 70578, Nanjing, China; 3 College of Veterinary Medicine, Northeast Agricultural University 12430, Harbin, China; 4 Northeast Science Observation Station for Animal Pathogen Biology, Ministry of Agriculture and Rural Affairs, Harbin, China; University of Michigan Medical School, Ann Arbor, Michigan, USA

**Keywords:** classical swine fever virus (CSFV), VPS34, PIK3C3, autophagy, autophagosome-lysosome fusion, UVRAG

## Abstract

Classical swine fever virus (CSFV) is a highly contagious and lethal pathogen that poses a major threat to the global swine industry. Despite its economic impact, no specific antiviral therapies are currently available, underscoring the urgent need to elucidate virus-host interactions for therapeutic innovation. In this study, we screened a glucose metabolism-targeted small-molecule library and identified Vps34-IN-1, a selective inhibitor of phosphatidylinositol 3-kinase class III (VPS34/PIK3C3), as a potent suppressor of CSFV replication in a dose-dependent manner. Time-of-addition experiments demonstrate that Vps34-IN-1 predominantly interferes with the late stage of the viral life cycle. Consistently, siRNA-mediated knockdown of VPS34 significantly impairs viral replication, confirming its role as a critical host dependency factor. Mechanistically, pharmacological inhibition or genetic silencing of VPS34 disrupts CSFV-induced autophagic flux. Notably, the CSFV non-structural protein p7 engages in a specific interaction with UVRAG, a pivotal constituent of the VPS34 complex II, and appears to potentiate VPS34-UVRAG complex assembly, thereby facilitating autophagosome-lysosome fusion. Collectively, these findings uncover an unappreciated role of VPS34 in sustaining CSFV replication and highlight its potential as a viable target for host-oriented antiviral intervention.

**IMPORTANCE:**

CSFV remains a major pathogen of global concern, causing severe disease in swine and incurring substantial economic losses in the pig industry. The absence of effective antiviral agents underscores the pressing need for host-targeted therapeutic strategies. In this study, we identified Vps34-IN-1, a selective inhibitor of VPS34, as a potent suppressor of CSFV replication in a dose-dependent manner. Remarkably, Vps34-IN-1 also exhibits potent inhibitory activity against other economically important swine viruses, including BVDV, PRV, and PEDV, demonstrating its potential as a broad-spectrum antiviral agent. Knockdown experiments further validated VPS34 as an essential host factor required for CSFV propagation. Mechanistically, the viral p7 protein engages in a specific interaction with UVRAG, a pivotal constituent of the VPS34 complex II, thereby potentially augmenting VPS34-UVRAG complex assembly and facilitating autophagosome-lysosome fusion. These findings delineate VPS34 as a compelling host-oriented antiviral target and open new therapeutic avenues for the control of CSF and other economically significant swine viral diseases.

## INTRODUCTION

Classical swine fever (CSF) was first identified in 1833 in Ohio, USA. Both domestic pigs and wild boars are susceptible to infection (1). Clinically, CSF is characterized by high fever, widespread hemorrhages, and immune suppression, with both high morbidity and mortality rates (2, 3). This severely restricts the development of the global swine industry. The causative agent of CSF, classical swine fever virus (CSFV), is an RNA, single-stranded, enveloped virus belonging to the *Pestivirus* genus within the *Flaviviridae* family. The CSFV genome is approximately 12.3 kb in size, containing two untranslated regions (UTRs) and a single open reading frame (ORF). The ORF encodes a polyprotein that is processed into four structural proteins (Core, Erns, E1, and E2) and eight non-structural proteins (Npro, p7, NS2, NS3, NS4A, NS4B, NS5A, and NS5B). These proteins play crucial roles in cellular invasion, pathogenicity, and immune evasion of CSFV (4–6). Currently, there are no specific antiviral drugs available against CSFV. Biosecurity and vaccination remain the cornerstone strategies for the prevention and control of CSFV. Despite the implementation of various biosecurity and vaccination protocols to curb outbreaks, eradicating CSF remains challenging in regions where the disease is endemic or re-emerges (7, 8). Considering the inherent limitations of current CSFV control measures, such as delays in vaccine-induced protection, gaps in field immunization, and challenges in containing wildlife-borne transmission, developing effective antiviral drugs for CSFV represents a critical complementary strategy. It not only helps fill the gaps left by vaccination-based approaches in specific scenarios but also provides a more scalable, practical alternative to costly interventions like monoclonal antibody therapy, thereby enhancing the comprehensiveness and flexibility of overall CSFV disease control. Notably, as CSFV is a representative member of the *Flaviviridae* family, antiviral strategies developed against CSFV may provide mechanistic insights and translational value applicable to other clinically significant *Flaviviridae* viruses, including Hepatitis C virus (HCV), Dengue virus (DENV), Zika virus (ZIKV), Japanese encephalitis virus (JEV), and Bovine viral diarrhea virus (BVDV).

Viral invasion of the host generally proceeds through a series of stages: attachment, binding, entry, uncoating, biosynthesis, assembly, and release (9). Among currently available antiviral drugs against CSFV, those targeting the viral life cycle predominate and have demonstrated protective efficacy across diverse CSFV genotypes (4). However, prolonged treatment may drive the emergence of drug-resistant and mutant strains, thereby diminishing therapeutic efficacy. In contrast, host-targeted antiviral approaches not only offer broader-spectrum antiviral efficacy but also effectively mitigate the risk of drug resistance (10, 11). Typically, *Flaviviruses* exploit host molecular mechanisms to create favorable microenvironments for replication (12). During infection, *Flaviviruses* modulate multiple host metabolic pathways. In the initial phase of infection, viral proteins interact with host proteins to regulate key metabolic pathways, including glucose, lipid, and nucleotide metabolism, thereby promoting viral replication and subsequent maturation (12–15). Understanding the interplay between viral replication and host metabolism provides a strong theoretical foundation for antiviral strategies. Therefore, this study employed a glucose metabolism-targeted compound library to screen for candidates with potent anti-CSFV activity and subsequently investigated their underlying antiviral mechanisms. The findings provide novel insights into the development of innovative therapeutic targets against CSFV and other *Flaviviridae* viruses.

Vacuolar protein sorting 34 (VPS34/PIK3C3) is the sole class III PI3K catalytic kinase in mammals, playing a critical role in autophagy and vesicular trafficking. Its N-terminal C2 domain mediates binding to Beclin 1 (16), while its C-terminal catalytic domain contains two key loop structures: one facilitates binding of the phosphatidylinositol (PI) substrate, and the other transfers the γ-phosphate group from ATP to PI, generating phosphatidylinositol 3-phosphate (PI3P) (17). PI3P serves as an essential signaling molecule required for membrane trafficking and autophagosome formation. VPS34, p150, and Beclin 1 are core components of the VPS34 complex, which regulates multiple physiological processes through interactions with various proteins (18–20). VPS34 complex I, comprising ATG14-VPS34-p150-Beclin 1, is localized within the endoplasmic reticulum (ER) and plays a pivotal role in initiating autophagy, thereby promoting the formation of autophagosomes (21). In contrast, complex II, consisting of UVRAG-VPS34-p150-Beclin 1, is situated in endosomes and lysosomes, where it is crucial for regulating membrane trafficking and vesicle transport (16). Additionally, complex II supports the late stages of autophagy by mediating autophagosome fusion with late endosomes/lysosomes, facilitating the degradation of autophagic substrates (22). In light of these functions, the role of VPS34 in viral infections has garnered significant attention. HCV NS4B orchestrates the recruitment of the Rab5-VPS34 complex to activate autophagic flux, thereby enhancing viral genome replication and virion biogenesis (23). A previous study has indicated that VPS34 is involved in both the replication of the HCV genome and the induction of autophagy, with the virus exploiting autophagic vesicles as sites for its genome synthesis (24). Furthermore, VPS34 aids Enteroviruses (EVs) in bypassing the intermediate capsid stage, thereby facilitating viral assembly (25). Infectious bronchitis virus (IBV) activates VPS34 complex I-induced autophagy to enhance viral proliferation (26). It has been demonstrated that CSFV NS5A disrupts the interaction between PP2A and Beclin 1 while promoting the association of PP2A with DAPK3. This redistribution enables PP2A to dephosphorylate and activate DAPK3, which, in turn, phosphorylates Beclin 1. The phosphorylated Beclin 1 subsequently enhances the activity of the VPS34 complex, thereby initiating autophagy and fostering an intracellular environment conducive to viral replication (27, 28). Interestingly, foot-and-mouth disease virus (FMDV) induces autophagosomes during cell entry via a VPS34-independent pathway (29). Overall, VPS34 plays a crucial role at multiple stages of autophagy, making it indispensable for viral replication and a highly attractive host target for the development of antiviral drugs.

Currently, the role of VPS34 during CSFV infection remains unclear. This study elucidated the molecular mechanism by which VPS34 regulated CSFV replication. Our results demonstrated that Vps34-IN-1 exhibited antiviral activity against multiple viruses, including CSFV, BVDV, Pseudorabies virus (PRV), and Porcine epidemic diarrhea virus (PEDV), underscoring VPS34 as a promising target for broad-spectrum antiviral intervention. Further investigation revealed that Vps34-IN-1 did not affect viral binding or entry, prompting additional investigation into the role of VPS34 in viral replication. The CSFV non-structural protein p7 interacted with UVRAG, and this association was thought to enhance the UVRAG-VPS34 interaction, thereby promoting autophagosome-lysosome fusion and accelerating autophagic flux to facilitate viral replication. In conclusion, these findings clarify the pivotal role of VPS34 in CSFV-induced autophagy and provide new insights for the development of antiviral therapies against CSFV.

## RESULTS

### Screening for anti-CSFV drugs from a glucose metabolism compound library

**Fig 1:**
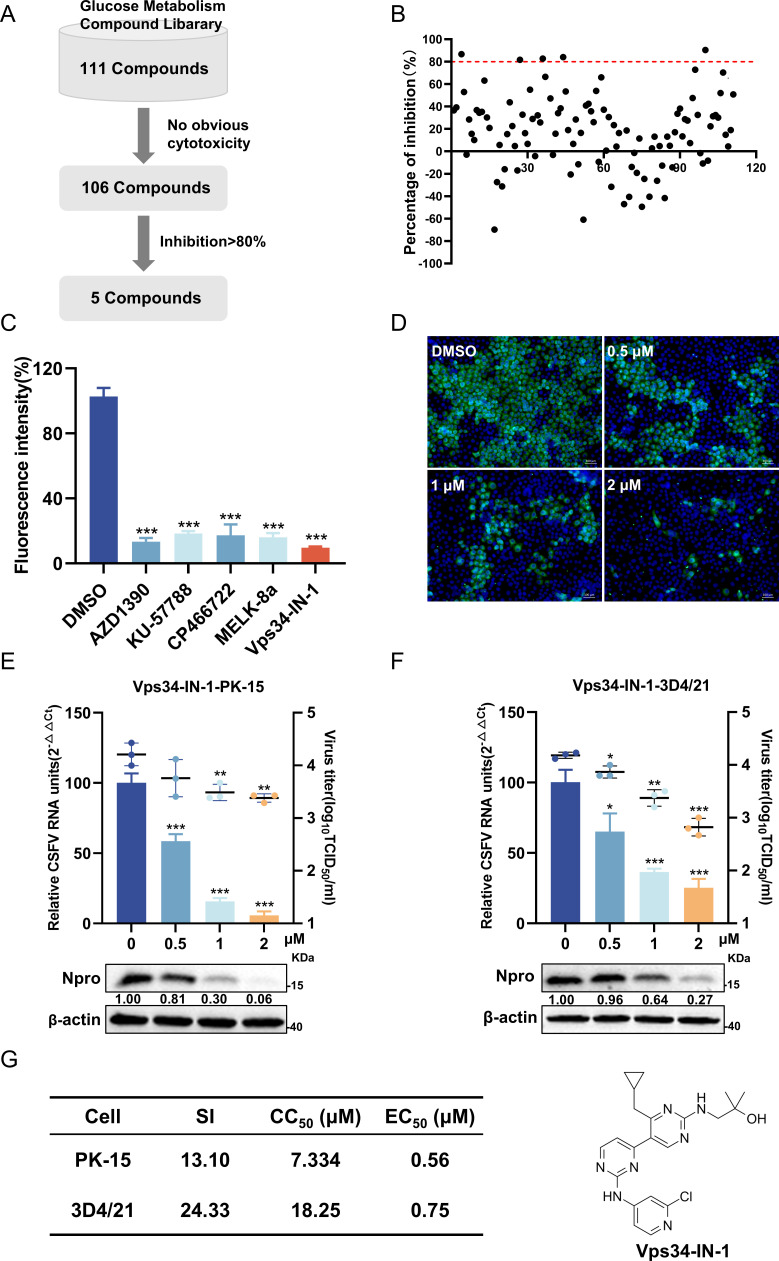
Screening of anti-CSFV compounds from a glucose metabolism library. (**A**) Screening process for antiviral compounds. (**B**) Inhibition effects of the compounds. Each dot represents the inhibition rate of a compound (1 µM) against CSFV(Multiplicity of infection [MOI] = 1). (**C**) The fluorescence intensity of CSFV E2 for the five compounds screened against CSFV was expressed as relative fluorescence intensity. (**D**) Different concentrations of Vps34-IN-1 were added to cells after infection with CSFV (MOI = 1). At 24 hours post-infection (hpi), cells were fixed and stained with mouse anti-CSFV E2 antibody (green) and observed by fluorescence microscopy. Scale bars = 100 µm. (**E and F**) The inhibition effect of Vps34-IN-1 was dose-dependent. PK-15 or 3D4/21 cells infected with CSFV (MOI = 1) were treated with Vps34-IN-1 for 24 h, harvested, and subjected to RT-qPCR, Western blotting, and virus titration. Protein expressions were calculated by determining the ratio of the protein to β-actin using ImageJ 7.0 software. (**G**) CC_50_, EC_50_, and selectivity index (SI = CC_50_/EC_50_) values of Vps34-IN-1 against CSFV on PK-15 and 3D4/21 cells. Data are presented as the mean ± SD from three independent experiments. *p < 0.05, **p < 0.01, ***p < 0.001.

CSFV infection induces significant metabolic alterations in the host, leading to the reprogramming of key metabolic pathways, including glucose, lipid, and nucleotide metabolism (30). These changes not only disrupt normal host metabolism but also create a favorable environment for viral replication. In our previous studies, compound library screening identified several molecules targeting lipid and nucleotide metabolism that exhibited strong inhibitory effects on CSFV replication (31, 32). Based on these findings, the current study aimed to identify novel antiviral targets for CSFV by screening a glucose metabolism compound library, comprising 111 compounds (Fig. 1A). Initially, PK-15 cells were used to assess cytotoxicity through the CCK-8 assay. Five compounds with high cytotoxicity significantly reduced cell viability and were therefore excluded from further analysis. The remaining 106 compounds were subsequently evaluated for antiviral efficacy. Indirect immunofluorescence assay (IFA) was performed to assess viral proliferation inhibition, revealing that five compounds achieved over 80% inhibition of CSFV proliferation. Among them, Vps34-IN-1 exhibited the strongest effect, with a 90.4% inhibition rate (Fig. 1B and C). To further investigate its antiviral activity, IFA was conducted at 24 hours post-treatment (hpt), demonstrating a dose-dependent reduction in CSFV E2 protein expression following Vps34-IN-1 treatment (Fig. 1D). Additionally, PK-15 and 3D4/21 cells treated with Vps34-IN-1 were collected for Western blotting and RT-qPCR. In alignment with our expectations, Vps34-IN-1 reduced CSFV Npro expression by 94.0% and 73.0%, and decreased RNA copies by 94.3% and 74.9% compared to the untreated group. Meanwhile, increasing concentrations of Vps34-IN-1 resulted in a gradual reduction in viral titers, with a statistically significant difference observed relative to the control group (Fig. 1E and F). To evaluate the pharmacological safety and therapeutic potential of Vps34-IN-1, the 50% cytotoxic concentration (CC_50_) and 50% effective concentration (EC_50_) were experimentally determined in PK-15 and 3D4/21 cells. The selectivity index (SI) was subsequently calculated as SI = CC_50_/EC_50_, providing an assessment of the compound’s therapeutic window. The results demonstrated that Vps34-IN-1 exhibited SI exceeding 10 in both cell lines (Fig. 1G), indicating a favorable safety profile and highlighting its potential for clinical application. In summary, these data identify Vps34-IN-1 as a potent and effective inhibitor of CSFV replication.

### Broad-spectrum antiviral activities of Vps34-IN-1

**Fig 2:**
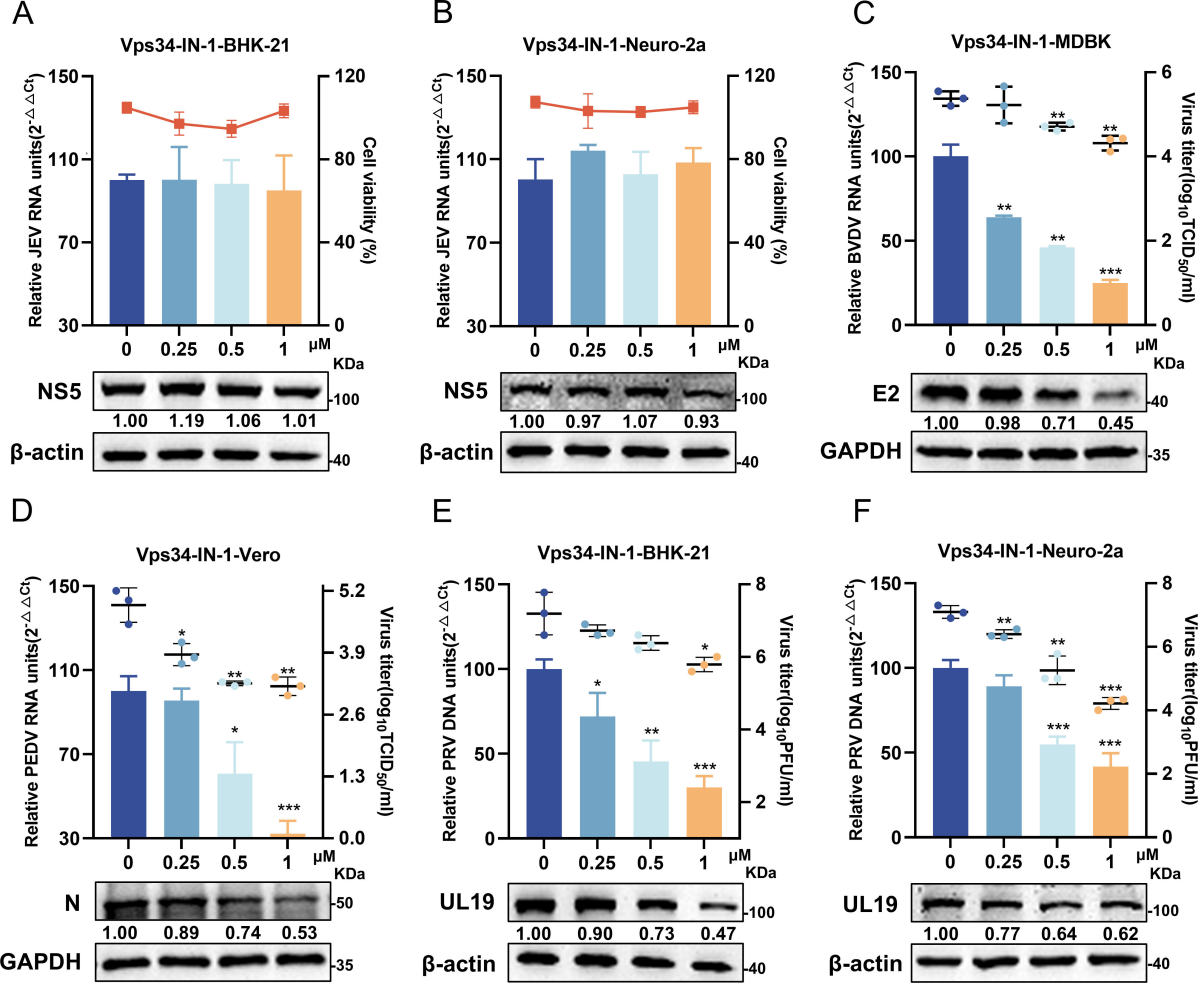
The broad-spectrum antiviral activities of Vps34-IN-1. (**A and B**) BHK-21 or Neuro-2a cells were infected with JEV (MOI = 0.1) and then treated with DMSO or increasing concentrations of Vps34-IN-1 for 24 h. Viral replication was assessed by Western blotting and RT-qPCR. The effects of the indicated compounds on cell cytotoxicity were quantified using the CCK-8 assay. (**C and D**) MDBK and Vero cells were respectively infected with BVDV (MOI = 0.5) (**C**) and PEDV (MOI = 0.1) (**D**), treated with DMSO or varying concentrations of Vps34-IN-1 for 24 h, harvested, and then subjected to RT-qPCR, Western blotting, and virus titration. (**E and F**) BHK-21 or Neuro-2a cells were infected with PRV (MOI = 0.1) and then treated with DMSO or different concentrations of Vps34-IN-1 for 24 h. Cells were harvested and subjected to RT-qPCR, Western blotting, and plaque assays. Protein expressions were quantified by calculating the ratio of protein to β-actin/GAPDH using ImageJ 7.0 software. Data are presented as the mean ± SD from three independent experiments. *p < 0.05, **p < 0.01, ***p < 0.001.

Recently, several specific inhibitors targeting VPS34 have been developed (33), with studies demonstrating their ability to suppress viral infections both *in vitro* and *in vivo*. Notably, Vps34-IN-1 has been reported to inhibit SARS-CoV-2 (34) and IBV (26) *in vitro*, while PIK-III effectively suppresses EV71 replication (35). To explore the broad-spectrum antiviral potential of Vps34-IN-1, cells were infected with multiple viruses, including JEV, BVDV, PRV, and PEDV, followed by the treatment with Vps34-IN-1. At 24 hpi, cells were harvested and subjected to RT-qPCR and Western blotting. The results showed that Vps34-IN-1 had no significant inhibitory effects on JEV replication (Fig. 2A and B). However, it exerted a dose-dependent inhibitory effect on BVDV replication, significantly suppressing the synthesis of viral RNA and protein expression, as well as virion production (Fig. 2C). These results suggest that Vps34-IN-1 may be a broad-spectrum antiviral agent against *Flaviviridae*. Considering the pronounced inhibitory effect of Vps34-IN-1 on SARS-CoV-2, we further assessed its antiviral activity against PEDV, another member of the *Coronaviridae* family. Vps34-IN-1 administration not only attenuated viral N protein expression by 47% and reduced viral mRNA abundance by 67.7% but also significantly suppressed viral titers, thereby reinforcing its efficacy in inhibiting viral replication (Fig. 2D). In addition to the aforementioned RNA viruses, Vps34-IN-1 also exhibited significant inhibitory effects on PRV in both cell lines. Vps34-IN-1 profoundly attenuated PRV replication across both cell lines. Specifically, UL19 protein expression was reduced by 53.0% and 38.0%, while viral mRNA copy numbers declined by 70.0% and 58.2%, respectively. In addition, Vps34-IN-1 markedly impaired the generation of infectious progeny virions (Fig. 2E and F). In conclusion, these results underscore that Vps34-IN-1 exhibits a significant inhibitory effect against a variety of viruses, including *Flaviviruses*, *Herpesviruses,* and *Coronaviruses*, suggesting its potential as a broad-spectrum antiviral candidate.

### Vps34-IN-1 affects the late stage of the viral life cycle

**Fig 3:**
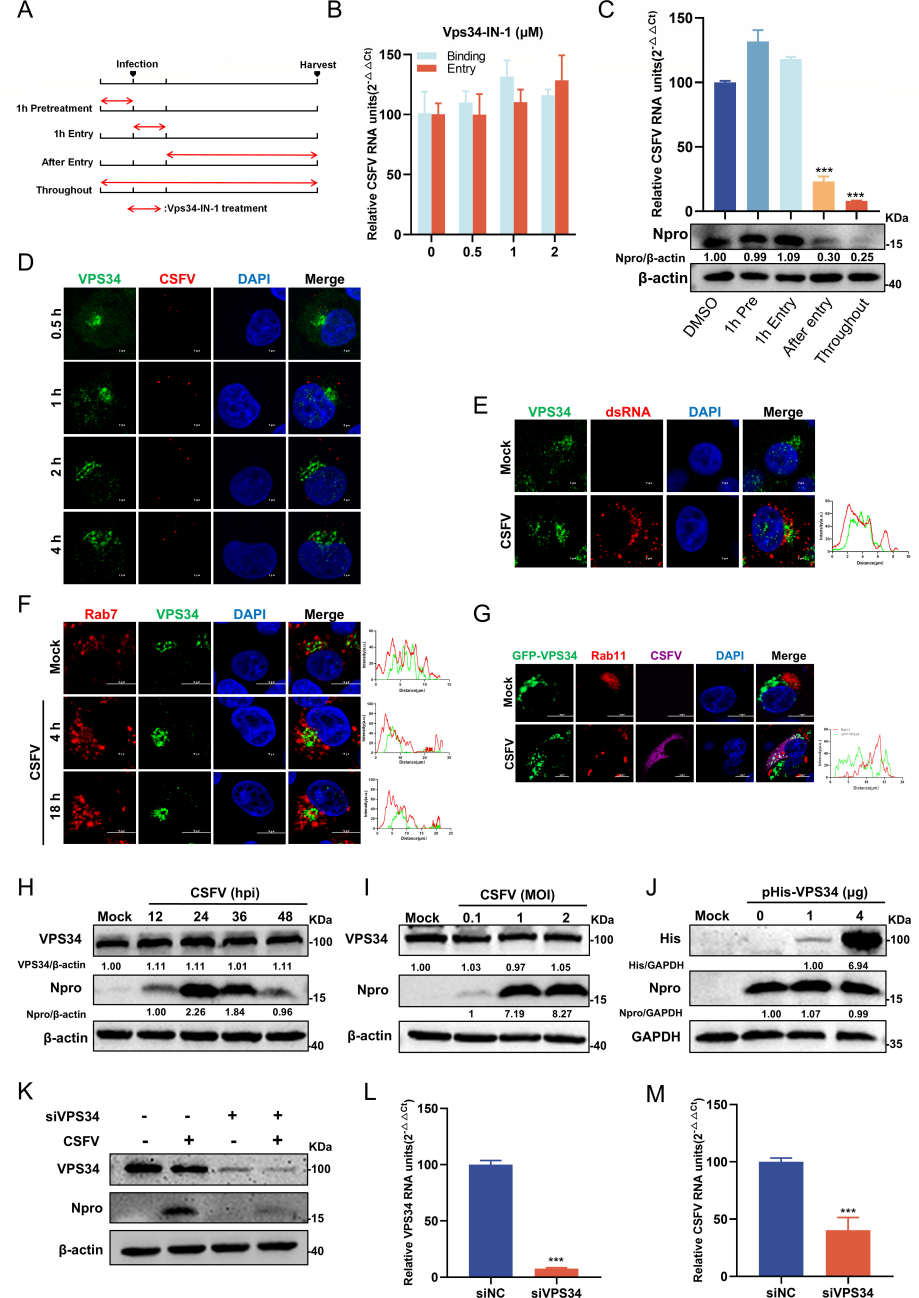
The significant effect of Vps34-IN-1 on the late stage of CSFV infection. (**A**) Schematic illustration of the time-of-addition assay. (**B**) PK-15 cells were treated with varying concentrations of Vps34-IN-1 and infected with CSFV (MOI = 10) for 1 h at 4°C, followed by incubation at 37°C for either 0 hpi (binding) or 1 hpi (entry). Cells were harvested and analyzed by RT-qPCR to measure viral mRNA copies. (**C**) The inhibitory effect of Vps34-IN-1 at specific stages of the viral life cycle was evaluated using a time-of-addition assay, as described below: (i) 1-h pretreatment: cells were treated with the indicated concentration (1 µM) of Vps34-IN-1 for 1 h before infection with CSFV (MOI = 1); (ii) 1-h entry: cells were treated with a mixture of the indicated Vps34-IN-1 and CSFV (MOI = 1) for 1 h; (iii) Post-entry: cells were treated with the indicated Vps34-IN-1 after infection with CSFV; (iv) Throughout: cells were treated with the indicated Vps34-IN-1 throughout the entire process. Treated cells were collected at 24 hpi and analyzed by RT-qPCR and Western blotting to assess viral replication. (**D**) The PK-15 cells infected with CSFV (MOI = 10) for 1 h at 4℃ were transferred to a 37℃ incubator. Samples were collected at 0.5, 1, 2, and 4 hpi, fixed, and stained with rabbit anti-VPS34 antibody and mouse anti-E2 antibody for confocal microscopy. (**E**) PK-15 cells infected with CSFV (MOI = 1) for 24 h were fixed and stained with rabbit anti-VPS34 antibody and mouse anti-dsRNA antibody for confocal microscopy. Co-localization analysis was performed using ImageJ 7.0 software. (**F**) PK-15 cells were infected with CSFV (MOI = 10) for 1 h at 4°C, then transferred to a 37°C incubator. Samples were collected at 4 and 18 hpi, fixed, and stained with rabbit anti-VPS34 antibody and mouse anti-Rab7 antibody for confocal microscopy. Scale bars = 10 µm. (**G**) PK-15 cells were transfected with pGFP-VPS34 and then infected with CSFV (MOI = 1). At 24 hpi, cells were fixed and stained with rabbit anti-Rab11 antibody and mouse anti-E2 antibody. Scale bars = 10 µm. (**H**) PK-15 cells infected with CSFV (MOI = 1) were collected at the indicated time points and analyzed by Western blotting using rabbit anti-Npro antibody. (**I**) PK-15 cells infected with varying MOIs of CSFV were collected at the indicated time points and analyzed by Western blotting. (**J**) PK-15 cells transfected with pHis-VPS34 (1 and 4 µg) were infected with CSFV (MOI = 1). At 24 hpi, cells were collected and examined by Western blotting. (**K through M**) PK-15 cells transfected with siNC or siVPS34 for 24 h were infected with CSFV (MOI = 1). Cells were collected and analyzed by RT-qPCR and Western blotting. Protein expressions were quantified by calculating the ratio of protein to β-actin or GAPDH using ImageJ 7.0 software. Data are presented as the mean ± SD from three independent experiments. *p < 0.05, **p < 0.01, ***p < 0.001.

Viral invasion of the host typically progresses through distinct stages, including attachment, entry, uncoating, biosynthesis, assembly, and release. To precisely determine the specific stage at which Vps34-IN-1 exerts its antiviral effect, a time-of-addition assay was performed (Fig. 3A). Cells were pre-treated with different concentrations of Vps34-IN-1 for 1 h, followed by CSFV infection for 1 h at 4℃, and then incubated at 37℃ at either 0 hpi (binding) or 1 hpi (entry). The results showed that pre-treatment of Vps34-IN-1 with a gradient concentration did not affect the binding or entry of CSFV (Fig. 3B). To further assess its role during infection, Vps34-IN-1 (1 µM) was added at different time points before or during infection. After 1 h pre-treatment, no significant changes in viral mRNA or protein expression were detected during the binding or entry. However, continuous treatment throughout the replication phase and post-entry treatment reduced viral mRNA copies by over 76.8% and decreased Npro protein expression by more than 70% (Fig. 3C). These findings indicate that Vps34-IN-1 specifically inhibits the replication phase of CSFV, rather than interfering with viral binding or entry. To further confirm the role of VPS34 in viral replication, a confocal microscopy assay was performed on cell samples collected at the indicated time points. No significant co-localization was observed between VPS34 and CSFV virions (Fig. 3D), suggesting that VPS34 was not directly engaged in early infection. Based on this, we hypothesized that VPS34 functioned during the replication phase of CSFV life cycle. To investigate its interaction with the viral replication complex, cells were infected with CSFV and stained with indicated antibodies against dsRNA and VPS34. The absence of discernible co-localization between VPS34 and dsRNA implied that VPS34 did not participate directly in the replication of the CSFV genome (Fig. 3E). To further assess the role of VPS34 in CSFV infection, confocal microscopy was employed to examine its co-localization with Rab7 and Rab11, two key regulators of endosomal trafficking. Confocal microscopy revealed negligible co-localization of Rab7 or Rab11 with VPS34 during CSFV infection (Fig. 3F and G). Next, to evaluate whether CSFV infection modulates VPS34 expression, endogenous protein expression was assessed at various MOIs and time points by Western blotting. The results showed that VPS34 protein expression remained unchanged following CSFV infection (Fig. 3H and I). Similarly, gradient overexpression of pHis-VPS34 had no detectable effect on viral replication (Fig. 3J), confirming that CSFV did not regulate VPS34 protein abundance. Furthermore, to determine whether VPS34 is essential for CSFV replication, cells were transfected with siRNA targeting VPS34 or a negative control siRNA (siNC) for 24 h and subsequently infected with CSFV (MOI = 1). Cells were then harvested and subjected to RT-qPCR and Western blotting. VPS34 knockdown resulted in a more than 50% reduction in Npro protein and viral mRNA expressions (Fig. 3K through M), demonstrating that VPS34 played an indispensable role in the CSFV life cycle. Collectively, these results confirm that VPS34 is required for CSFV replication but does not directly participate in genome synthesis.

### VPS34 is involved in CSFV-induced autophagy

**Fig 4:**
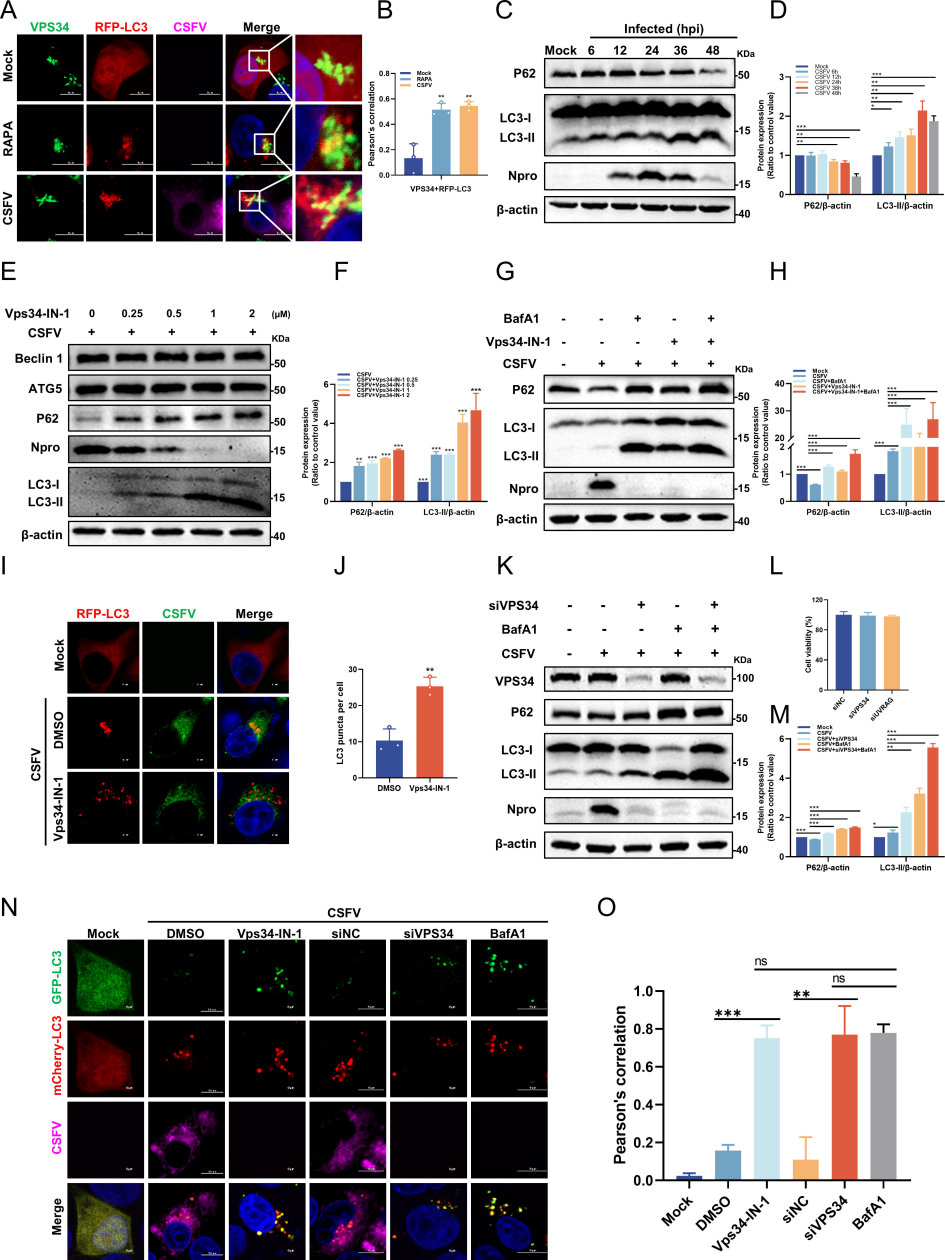
VPS34 is involved in CSFV-induced autophagy. (**A**) Cells transfected with the RFP-LC3 plasmid were treated with RAPA (100 nM) or infected with CSFV (MOI = 1) for 24 h. After fixation, cells were stained with rabbit anti-VPS34 antibody and mouse anti-E2 antibody. Scale bars = 10 µm. (**B**) The co-localization analysis was expressed as Pearson’s correlation coefficient. (**C through H**) PK-15 cells infected with CSFV (MOI = 1) were either collected at the indicated time points (**C and D**), treated with different concentrations of Vps34-IN-1 (**E and F**), or treated with Vps34-IN-1 (1 μM), BafA1 (0.2 μM), or both (**G and H**). Cells were harvested at 24 hpi and subjected to Western blotting using rabbit anti-Npro antibody, rabbit anti-P62 antibody, and rabbit anti-LC3 antibody. The expression of protein was quantified using ImageJ 7.0 software. (**I and J**) PK-15 cells transfected with RFP-LC3 plasmid were infected with CSFV (MOI = 1) and incubated with Vps34-IN-1 for 24 h, then fixed and stained with mouse anti-E2 antibody for confocal microscopy. (**K through M**) PK-15 cells transfected with siVPS34 or siNC were inoculated with CSFV (MOI = 1), followed by the addition of BafA1 (0.2 μM). Cells were harvested and subjected to Western blotting with the indicated antibodies. Protein expressions were quantified by calculating the ratio of protein to β-actin using ImageJ 7.0 software. Cell viability following transfection with all siRNAs was assessed using the CCK-8 assay. (**N**) Cells treated with DMSO or Vps34-IN-1 (1 µM), or transfected with siVPS34 or siNC, were transfected with mCherry-GFP-LC3 for 12 h and then infected with CSFV (MOI = 1). Cells treated with BafA1 (0.2 µM) served as a positive control. At 24 hpi, the cells were fixed with 4% paraformaldehyde and detected by confocal microscopy. The right panels show the red (mCherry-LC3) and green (GFP-LC3) pixel intensity. Scale bars = 10 µm. (**O**) The co-localization analysis was expressed as Pearson’s correlation coefficient. Data are presented as the mean ± SD from three independent experiments. *p < 0.05, **p < 0.01, ***p < 0.001, ns: no significance.

VPS34 catalyzes the generation of PI3P (36) and regulates endosomal trafficking and autophagy through dynamic interactions with regulatory complex subunits. It has been well established that CSFV activates a complete autophagy process within host cells to facilitate viral replication (37). Based on these data, we postulate that VPS34 may modulate CSFV replication by regulating autophagy. To evaluate this hypothesis, a confocal microscopy assay was employed to compare the spatial co-localization of VPS34 with LC3 between CSFV-infected and uninfected cells, with rapamycin (RAPA) treatment serving as a positive control for autophagy induction. As shown in Fig. 4A, prominent co-localization between VPS34 and LC3 puncta was observed in CSFV-infected cells. The subsequent co-localization coefficient analysis was in good agreement with these interactions (Fig. 4B). To further delineate the autophagic response elicited by CSFV, temporal profiling of autophagy-related markers was performed during infection. PK-15 cells infected with CSFV were harvested at various time points for Western blotting. The results demonstrated a progressive elevation of LC3-II and a time-dependent decline in P62 expression, confirming that CSFV potently activated complete autophagic flux in host cells (Fig. 4C and D). Recent studies have highlighted the potential of VPS34 inhibitors to modulate autophagic flux (33). To further explore this phenomenon, cells were treated with increasing concentrations of Vps34-IN-1, and autophagy-related markers were assessed via Western blotting, followed by quantitative analysis. Intriguingly, VPS34 inhibition led to a marked increase in the LC3-II and elevated P62 protein expression (Fig. 4E and F). The autophagosome accumulation observed following Vps34-IN-1 treatment, which was presumed to result from a blockade in downstream autophagic progression, was further investigated. To verify this possibility, autophagic flux was assessed using bafilomycin A1 (BafA1), an inhibitor that blocks autophagosome-lysosome fusion. Co-treatment with Vps34-IN-1 and BafA1 resulted in a notable upregulation in LC3-II and P62 levels compared to cells treated with BafA1 alone (Fig. 4G and H). Additionally, confocal microscopy was performed to observe the effects of Vps34-IN-1 or DMSO treatment on LC3 in infected cells. Compared to the DMSO control, Vps34-IN-1 treatment resulted in a significant accumulation of LC3 puncta (Fig. 4I and J), indicating that VPS34 dysfunction did not impair autophagy initiation but instead induced changes in autophagic flux. To substantiate these observations, siVPS34-transfected cells were infected with CSFV (MOI = 1) and subsequently treated with BafA1. At 24 hpi, cells were harvested and subjected to Western blotting. As expected, VPS34 knockdown significantly reduced the expression of CSFV Npro protein, reinforcing its role in facilitating CSFV replication. Notably, relative to the control group, VPS34 knockdown induced a marked upregulation of LC3-II and P62 expression, indicating a pronounced disruption of autophagic flux, while cytotoxicity assessment confirmed that the interference primers used in this study exhibited no detectable cytotoxic effects (Fig. 4K through M). To directly evaluate the role of VPS34 in autophagy flux, cells were treated with the Vps34-IN-1 or transfected with siVPS34, followed by transfection with the mCherry-GFP-LC3 tandem construct, which differentiates autophagosomes (yellow puncta) from autolysosomes (red puncta). Subsequently, PK-15 cells were infected with CSFV (MOI = 1), while cells treated with BafA1 served as a positive control. As shown in Fig. 4N, CSFV-infected cells exhibited red puncta fluorescence, indicative of complete autophagy induction. Conversely, VPS34 inhibition or knockdown resulted in a transition from red to partially yellow fluorescence, mirroring the effect of BafA1 treatment. Subsequent co-localization coefficient analysis further validated this observation (Fig. 4O), suggesting that both VPS34 inhibition and knockdown impaired autophagy flux. In conclusion, this study delineates the pivotal role of VPS34 in mediating CSFV-induced autophagy and demonstrates that its functional impairment disrupts autophagic flux during viral infection.

### VPS34 plays a key role in the autophagosome-lysosome fusion stage of autophagy induced by CSFV

**Fig 5:**
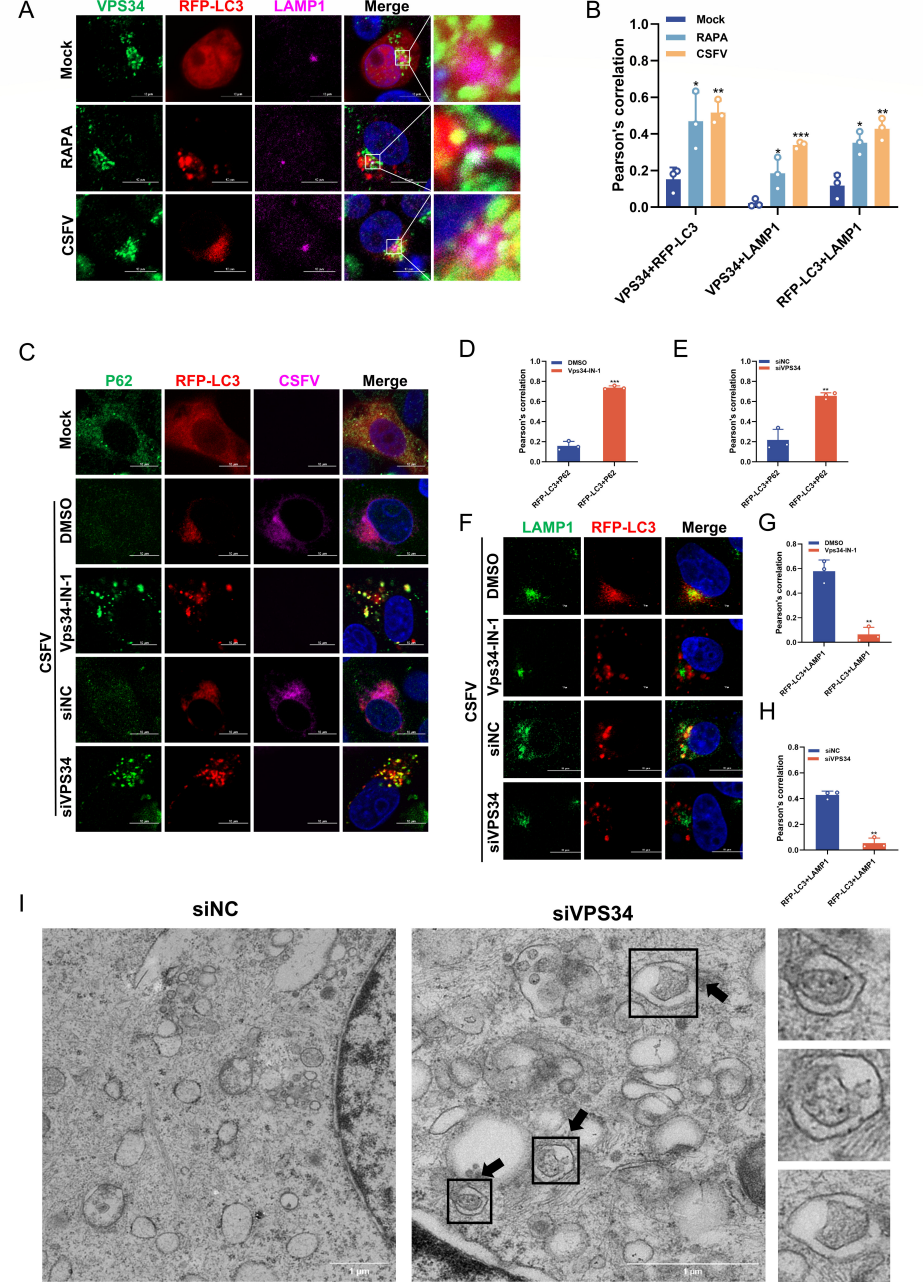
VPS34 plays a pivotal role in the fusion stage of autophagosomes with lysosomes in CSFV-induced autophagy. (**A**) PK-15 cells transfected with RFP-LC3 plasmid were treated with RAPA (100 nM) or infected with CSFV (MOI = 1) for 24 h, fixed, and stained with rabbit anti-VPS34 antibody and mouse anti-LAMP1 antibody. Scale bars = 10 µm. (**B**) The co-localization analysis was expressed as Pearson’s correlation coefficient. (**C through H**) Cells transfected with RFP-LC3 were infected with CSFV (MOI = 1) for 24 h. After fixation, cells were subjected to immunofluorescent staining using anti-P62 antibody (**C**) or anti-LAMP1 antibody (**F**). Scale bars = 10 µm. The co-localization analysis was expressed as Pearson’s correlation coefficient (**D and E, G and H**). (**I**) Cells were transfected with siVPS34 or siNC and then infected with CSFV (MOI = 1), then harvested at 24 hpi and analyzed by TEM. Scale bar = 1 µm. Data are presented as the mean ± SD from three independent experiments.*p < 0.05, **p < 0.01, ***p < 0.001.

Studies have illustrated that VPS34 plays a crucial role in multiple stages of autophagy, including autophagy initiation, autophagosome nucleation, and autophagosome-lysosome fusion, thereby regulating autophagic flux (18–20). Based on these findings, we hypothesized that VPS34 dysfunction would disrupt the autophagosome-lysosome fusion process. To further investigate this, a confocal microscopy assay was employed to examine the co-localization of VPS34, LC3, and LAMP1 following CSFV infection, with RAPA treatment serving as a positive control for autophagy induction. The results revealed that VPS34 co-localized with LC3 and LAMP1 during viral infection (Fig. 5A and B), mirroring the effects observed in the RAPA-treated group, which indicated its involvement in the autophagosome-lysosome fusion stage. A previous study has validated that P62 is present in autophagosomes and is degraded within the autolysosomes after autophagosome-lysosome fusion (37). To delineate the specific role of VPS34 in CSFV-induced autophagy, the co-localization of P62 with LC3 was examined via confocal microscopy. Relative to the control group, cells subjected to VPS34 inhibition or knockdown displayed a pronounced elevation in P62 fluorescence intensity, accompanied by a substantial increase in LC3-P62 co-localization (Fig. 5C through E). These findings suggest that VPS34 inhibition or depletion results in P62 accumulation within autophagosomes, indicative of impaired autophagosome-lysosome fusion. To interrogate the role of VPS34 in autophagosome-lysosome fusion during CSFV infection, the spatial co-localization of LC3 with LAMP1 was evaluated by confocal microscopy. Functional abrogation of VPS34, either through pharmacological inhibition or siRNA-mediated knockdown, substantially diminished co-localization of LC3 with LAMP1 (Fig. 5F). These results were further supported by quantitative co-localization coefficient analysis (Fig. 5G and H). Moreover, transmission electron microscopy (TEM) was performed to provide a more direct assessment of the impact of VPS34 knockdown on autophagic flux. In contrast to the siNC control, cells treated with siVPS34 primarily accumulated double-membrane autophagosomes, signifying impaired fusion with lysosomes (Fig. 5I). In summary, these data underscore that the loss of VPS34 function impairs the fusion of autophagosomes with lysosomes during CSFV-induced autophagy.

### CSFV non-structural protein p7 facilitates VPS34-UVRAG complex formation

**Fig 6:**
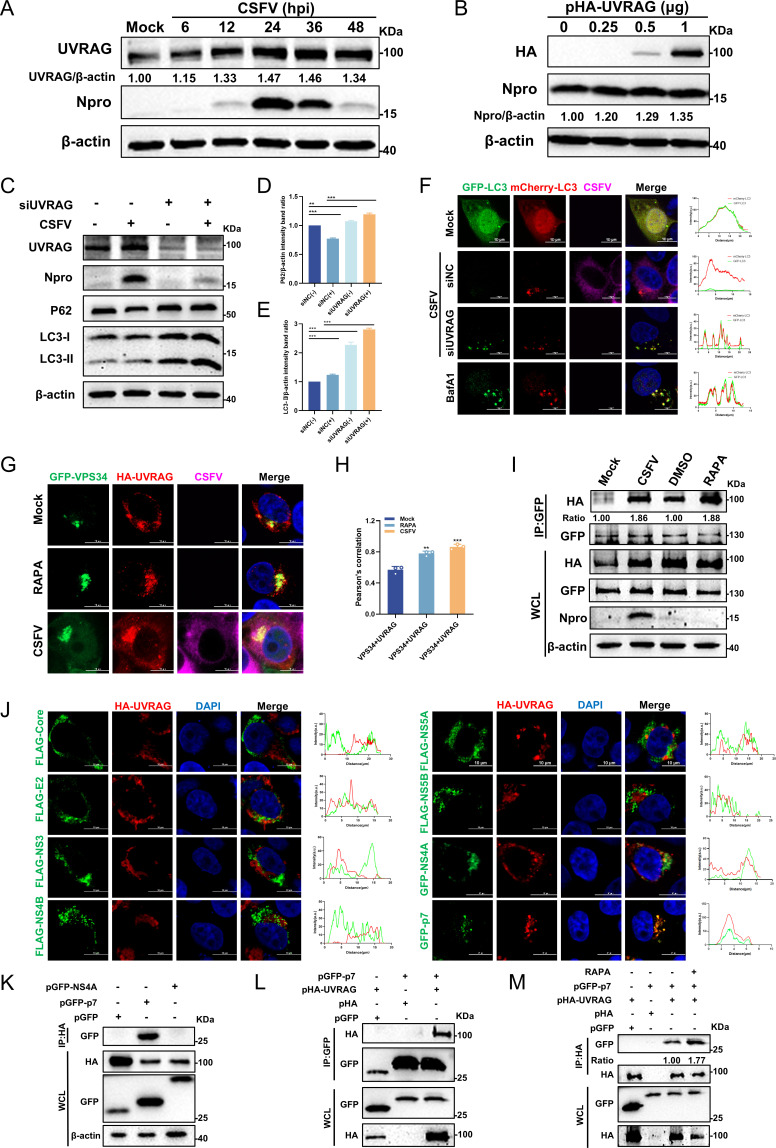
CSFV non-structural protein p7 facilitates VPS34-UVRAG complex formation. (**A**) PK-15 cells infected with CSFV (MOI = 1) were collected at the indicated time points and analyzed by Western blotting using rabbit anti-Npro antibody and rabbit anti-UVRAG antibody. (**B**) PK-15 cells transfected with varying concentrations of pHA-UVRAG were infected with CSFV (MOI = 1). At 24 hpi, cells were harvested and analyzed by Western blotting. (**C through E**) PK-15 cells transfected with siNC or siUVRAG for 24 h were then infected with CSFV (MOI = 1). Cells were collected and analyzed by Western blotting. β-actin was used as a loading control. (**F**) Cells transfected with siNC or siUVRAG were then transfected with mCherry-GFP-LC3 for 12 h and subsequently infected with CSFV (MOI = 1). Cells treated with BafA1 (0.2 µM) served as a positive control. (**G**) PK-15 cells co-transfected with pGFP-VPS34 and pHA-UVRAG plasmids were treated with RAPA or infected with CSFV (MOI = 1). At 24 hpi, the cells were fixed and stained with rabbit anti-HA antibody and mouse anti-E2 antibody. (**H**) The co-localization analysis was expressed as Pearson’s correlation coefficient. (**I**) Cells co-transfected with pHA-UVRAG and pGFP-VPS34 plasmids were infected with CSFV (MOI = 1). At 24 hpi, cells were harvested for Co-IP assay, and whole-cell lysates were subjected to Western blotting using rabbit anti-GFP antibody, rabbit anti-HA antibody, and rabbit anti-Npro antibody. The quantities of CSFV/Mock and RAPA/DMSO were used to calculate the grayscale using ImageJ 7.0 software. (**J**) PK-15 cells were co-transfected with pHA-UVRAG and different CSFV plasmids for 24 h. Cells were then fixed and stained with the indicated antibodies for analysis by confocal microscopy. Scale bars = 10 µm. Co-localization analysis was performed using ImageJ 7.0 software. (**K**) HEK-293T cells were co-transfected with pHA-UVRAG, pGFP-N1, pGFP-p7, or pGFP-NS4A for 24 h, harvested for Co-IP with anti-HA antibody, and the resulting proteins were analyzed by Western blotting with specific antibodies. (**L and M**) PK-15 cells were co-transfected with pHA-UVRAG, pGFP-N1, or pGFP-p7 for 24 h, harvested for Co-IP using anti-HA/GFP antibodies, and the resulting proteins were subjected to Western blotting with specific antibodies. Protein expression levels were quantified by calculating the ratio of protein to β-actin using ImageJ 7.0 software. Data are presented as the mean ± SD from three independent experiments. *p < 0.05, **p < 0.01, ***p < 0.001.

VPS34 assembles into distinct functional complexes through interactions with specific proteins. Among these, complex I and complex II share a core structure consisting of VPS34, VPS15, and Beclin 1, but differ in their associated regulatory subunits. Complex I is defined by its association with Atg14L, whereas complex II is distinguished by its interaction with UVRAG (38). The former primarily functions in the initiation and nucleation stages of autophagy, while the latter plays a pivotal role in endosomal trafficking and the autophagosome-lysosome fusion process (39, 40). To elucidate the role of UVRAG in CSFV infection, PK-15 cells were infected with CSFV (MOI = 1) and harvested at specified time intervals for Western blotting. The results indicated that UVRAG protein expression increased over time following CSFV infection (Fig. 6A). To further explore this relationship, cells were transfected with varying concentrations of pHA-UVRAG and infected with CSFV (MOI = 1). At 24 hpi, Western blotting results revealed that CSFV replication was enhanced in proportion to increasing pHA-UVRAG protein expression (Fig. 6B). Subsequently, endogenous UVRAG expression was knocked down via siRNA transfection, which significantly inhibited CSFV proliferation, indicating that UVRAG facilitated CSFV replication. Furthermore, UVRAG knockdown led to a significant elevation in the LC3-II and P62 protein expression (Fig. 6C through E). Additionally, mCherry-GFP-LC3 detection was performed to confirm the role of UVRAG in autophagic flux. UVRAG knockdown significantly enhanced yellow fluorescence, indicating that UVRAG depletion disrupted autophagic flux in CSFV-induced autophagy (Fig. 6F). Building upon these observations, we proposed that CSFV infection may enhance the physical interaction between VPS34 and UVRAG, thereby facilitating autophagosome-lysosome fusion efficiency. To investigate this, the co-localization of VPS34 and UVRAG was examined via confocal microscopy following CSFV infection. A significant enhancement in the co-localization between pEGFP-VPS34 and pHA-UVRAG was detected in both CSFV-infected cells and RAPA-treated positive controls, relative to the Mock group (Fig. 6G). Co-localization coefficient analysis further confirmed this observation (Fig. 6H). Consistently, a co-immunoprecipitation (Co-IP) assay was subsequently conducted to validate the interaction between VPS34 and UVRAG. The results revealed a pronounced enhancement of the VPS34-UVRAG association following CSFV infection, paralleling the effect observed with the autophagy inducer RAPA (Fig. 6I). A previous study has reported that the ORF3a protein of SARS-CoV-2 directly interacts with UVRAG to remodel the VPS34 complex, thereby disrupting autophagosome maturation and promoting viral persistence (41). In light of these findings, we sought to determine how CSFV exploits the VPS34 complex II to facilitate its replication. To this end, our study screened for CSFV proteins that interacted with UVRAG. Plasmids encoding various CSFV structural or non-structural proteins were transfected into PK-15 cells for 24 h, followed by a confocal microscopy assay to evaluate the co-localization of UVRAG with CSFV proteins. The results demonstrated that p7 co-localized with UVRAG (Fig. 6J), suggesting a potential interaction between them. A Co-IP assay was subsequently conducted to validate the interaction between p7 and UVRAG (Fig. 6K and L), which was found to be markedly enhanced upon treatment with the autophagy inducer RAPA (Fig. 6M). Collectively, our findings suggest that the CSFV non-structural protein p7 engages in a dynamic interaction with UVRAG, which may potentiate UVRAG’s association with VPS34, thereby promoting autophagosome-lysosome fusion and facilitating CSFV replication.

## DISCUSSION

CSF imposes substantial economic burdens on the global pig industry (42). At present, no effective specific antiviral drugs are available for CSFV, and biosecurity measures coupled with vaccination remain the primary strategies for prevention and control (7). This study does not aim to supplant existing preventive measures such as vaccination. Rather, we propose host-targeting compounds like Vps34-IN-1 as complementary interventions that address key limitations in current control strategies—particularly in scenarios involving subclinical infections, delayed onset of vaccine-induced protection, or inadequate immunization coverage in the field—by offering a targeted means to suppress viral transmission.

In our study, the IFA assay was employed to screen a glucose metabolism-targeted compound library, identifying Vps34-IN-1 as the most potent inhibitor. Vps34-IN-1 is a highly selective and specific VPS34 inhibitor that significantly suppresses VPS34 activity without affecting the activity of 340 protein kinases, including all Class I and Class II PI3K isoforms, as well as 25 lipid kinases (43). Previous studies have shown that Vps34-IN-1 exhibits antiviral activity *in vitro*, effectively inhibiting the replication of multiple viruses, including IBV (26) and SARS-CoV-2 (34). Our findings further expanded the antiviral research scope of VPS34 inhibitors, revealing that Vps34-IN-1 also effectively suppressed the replication of multiple viruses, including BVDV (*Flaviviridae*), PEDV (*Coronaviridae*), and PRV (*Herpesviridae*). These results collectively suggest that VPS34 inhibitors hold promise as broad-spectrum antiviral agents. VPS34 plays a crucial role in various stages of autophagy, making VPS34 inhibitors valuable tools for modulating autophagic processes (33). Since certain viruses exploit autophagy to facilitate their replication, targeting autophagy has emerged as a potential antiviral strategy (42, 44, 45). Previous reports have shown that JEV infection disrupts autophagic flux and compromises lysosomal function, which may account for the limited efficacy of Vps34-IN-1 in suppressing JEV replication observed in our experiments (46). Beyond its broad-spectrum antiviral activity, Vps34-IN-1 also offers several practical advantages for disease control. In contrast to monoclonal antibodies, which often encounter challenges related to large-scale production, cold-chain storage, and field deployment, Vps34-IN-1 demonstrates favorable attributes such as scalable chemical synthesis, ease of administration, and suitability for use in field settings. Although direct evidence from CSFV-infected porcine models is not yet available, its previously reported *in vivo* antiviral efficacy supports its promise as a lead compound for further development (26). Vps34-IN-1 remains in the early stages of preclinical investigation and requires additional optimization to improve potency, target specificity, and pharmacokinetic properties. Future efforts should prioritize structural refinement and systematic *in vivo* validation, including evaluation of viral load reduction, alleviation of clinical symptoms, and survival outcomes in infected animals. Collectively, these findings underscore the value of host-targeted small-molecule inhibitors such as Vps34-IN-1 as complementary antiviral strategies. When administered in conjunction with vaccination, such compounds may address critical gaps in existing control strategies, particularly in situations involving subclinical infections, delayed onset of vaccine-induced immunity, or insufficient immunization coverage in the field. This combined approach could enhance the overall effectiveness of CSFV management programs.

**Fig 7:**
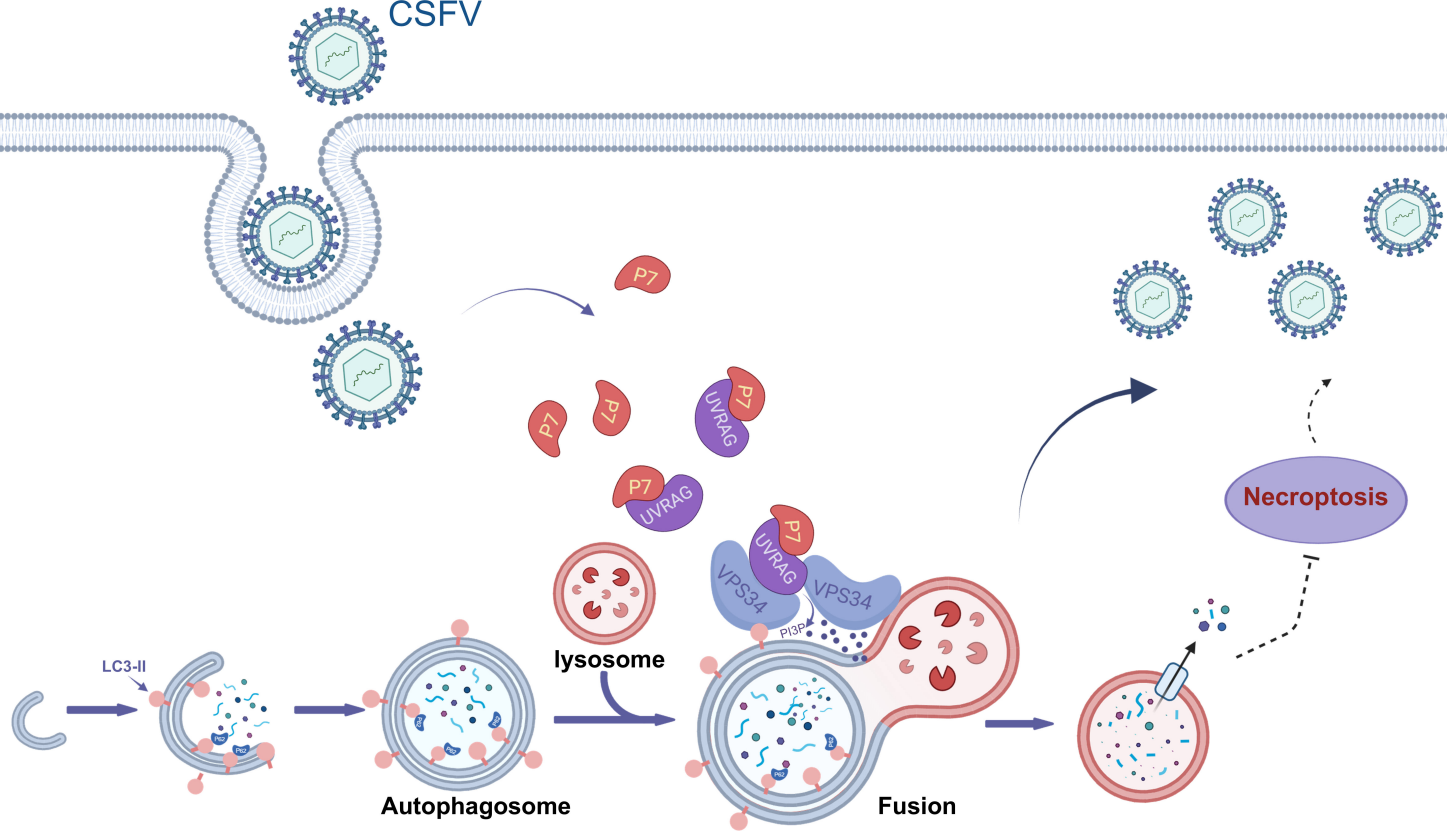
Schematic model depicting the role of VPS34 in the regulation of CSFV-induced autophagy. Following CSFV infection in PK-15 cells, the viral p7 protein may engage UVRAG, potentially enhancing VPS34-UVRAG complex formation. The resulting acceleration of autophagic flux, promoted by enhanced autophagosome-lysosome fusion, could, in turn, attenuate necroptosis, thereby fostering a cellular environment that supports persistent viral replication (created with BioRender.com).

The high specificity of Vps34-IN-1 highlights the critical role of VPS34 in CSFV replication. In contrast to EV71 infection, where VPS34 is recruited to replication organelles (ROs) (35), our study found no co-localization between VPS34 and dsRNA. However, VPS34 knockdown via siRNA significantly suppressed CSFV replication, indicating that VPS34 was essential for CSFV replication, albeit not through direct participation in viral genome replication. Accumulating evidence highlights the pivotal role of VPS34 in orchestrating endosomal and phagosomal trafficking, as well as in mediating autophagy initiation and autophagosome-lysosome fusion (47). It has also been demonstrated that CSFV infection induces complete autophagy in host cells, facilitating viral replication and particle release (48). Confocal microscopy assay in this study revealed pronounced co-localization of VPS34 with the autophagy marker LC3 following CSFV infection, indicating that VPS34 was actively involved in CSFV-induced autophagic processes. Interestingly, existing studies primarily emphasize the role of VPS34 in autophagy initiation. For instance, IBV enhances viral replication by activating VPS34 complex I to initiate autophagy (26), while the N protein of PPRV interacts with VPS34 complex I to induce autophagy (49). Additionally, Hepatitis B virus enhances its replication by promoting VPS34-mediated autophagy induction through RAB5A (50). However, the function of VPS34 in the late stage of autophagy remains limited. In this study, VPS34 was directly targeted, revealing its indispensable role during the late stage of CSFV-induced complete autophagy. VPS34 depletion disrupted autophagosome-lysosome fusion, thereby impairing autophagic flux. Previous studies reported that VPS34 complex II generates PI3P at the autophagosome-lysosome interface to facilitate their fusion (16, 22). Although the precise target specificity of Vps34-IN-1 has yet to be fully delineated, existing evidence suggests that the compound may preferentially act on complex II, as inferred from the selective inhibition of this complex by structurally analogous VPS34 inhibitors (e.g., SB02024) (33). The data demonstrate that both pharmacological inhibition and siRNA-mediated knockdown of VPS34 result in the accumulation of LC3-II and p62, a phenotypic signature indicative of impaired autophagic flux. This accumulation suggests that VPS34 suppression primarily disrupts the function of complex II, thereby obstructing autophagosome-lysosome fusion, rather than interfering with complex I-mediated autophagy initiation. Based on this observation, the investigation was subsequently directed toward UVRAG, a critical regulatory subunit of complex II. Further analysis of UVRAG revealed that its depletion markedly disrupted autophagic flux, underscoring its indispensable role in facilitating autophagosome maturation and lysosomal fusion. Building upon this, subsequent experiments demonstrated that the CSFV non-structural protein p7 dynamically associated with UVRAG following infection. This interaction may potentiate the assembly of the UVRAG-VPS34 complex, thereby promoting autophagosome-lysosome fusion, enhancing the efficiency of autophagic progression, and ultimately creating a cellular environment conducive to viral replication (Fig. 7). Although these findings support the existence of a functional axis involving p7, UVRAG, and VPS34, the mechanistic nature of this interaction remains to be fully elucidated, specifically, whether p7 directly recruits UVRAG to initiate complex formation or instead binds to pre-assembled complexes to stabilize their function. Such modulation of the host autophagy machinery by CSFV likely serves as a multifaceted survival strategy, whereby the enhancement of autophagic flux not only facilitates the clearance of damaged organelles that might otherwise compromise viral replication (51, 52), but also antagonizes necroptosis—a programmed cell death pathway integral to host antiviral defense—thereby enabling sustained viral persistence within the infected host (53). While our study has established VPS34 as a pivotal regulator in the late stage of CSFV-induced autophagy, additional evidence is required to determine whether the initiation and nucleation of autophagosomes are mediated through a VPS34-independent pathway. Remarkably, recent studies have revealed that ULK1 kinase facilitates lactate secretion by phosphorylating the glycolytic enzyme lactate dehydrogenase A at serine 196. Subsequently, lactate mediates the lactylation of VPS34 at lysine residues 356 and 781, a modification catalyzed by the acyltransferase KAT5/TIP60, which enhances VPS34 complex formation and its kinase activity, thereby promoting both autophagy and the endosome-lysosome degradation pathway (54). These findings underscore the intricate link between autophagy and glycolysis via VPS34, highlighting its central role in regulating both autophagic flux and cellular metabolism. Future investigations into the regulatory mechanisms of VPS34 within autophagy and metabolic networks will deepen our understanding of viral infections, cellular metabolism, and disease pathogenesis, paving the way for innovative therapeutic strategies against a range of viral diseases.

In summary, our study identified Vps34-IN-1 as a therapeutic candidate for combating CSFV infection and elucidated the underlying mechanistic role of VPS34 in CSFV replication. These findings provide the first comprehensive insight into the function of VPS34 in CSFV replication and highlight potential therapeutic targets for developing novel antiviral strategies against CSF.

## MATERIALS AND METHODS

### Virus, cells, plasmids, and antibodies

This study was conducted in strict compliance with the Chinese government and the university. All experimental activities were carried out in laboratories classified under the appropriate biosafety levels, as designated in the “Catalog of Human Infectious Pathogenic Microorganisms” and the “Classification Catalog of Animal Pathogenic Microorganisms,” and were performed in accordance with standardized operating procedures to ensure rigorous laboratory biosafety.

CSFV Shimen strain (GenBank accession number: AF092448), JEV NJ2008 strain (GenBank accession number: GQ918133), PRV SD2019 strain (GenBank accession number: OM959370), BVDV-2 strain (GenBank accession number: MG420995), and PEDV (HN2021, GenBank accession number: OR707084) were kept in the lab. Cells were cultured in Dulbecco’s modified Eagle’s medium (DMEM, GIBCO, Invitrogen, CA, USA) supplemented with 10% fetal bovine serum (FBS, GIBCO, Invitrogen, CA, USA), 0.2% NaHCO_3_, 100 µg/mL streptomycin, and 100 IU/mL penicillin (GIBCO, Invitrogen, CA, USA). The plasmids pFLAG-NS3, -NS4B, -NS5A, -NS5B, -E2, -Core were generated by cloning corresponding CSFV genes into the p3×FLAG-CMV-7.1 vector. The plasmid pEGFP-C1-NS4A was generated by cloning the corresponding CSFV NS4A gene into the pEGFP-C1 vector. The plasmid pEGFP-N1-p7 was generated by cloning the corresponding CSFV p7 gene into the pEGFP-N1 vector. The plasmid pmCherry-GFP-LC3 was kindly provided by Prof. Hongbo Zhou (Huazhong Agricultural University, Wuhan, China). The plasmid pRFP-LC3 was kindly provided by Prof. Boli Hu (Zhejiang University, Hangzhou, China). The plasmid pHis-VPS34 was kindly provided by Prof. Yingli Shang (Shandong Agricultural University, Taian, China). The construct pHA-UVRAG was purchased from Addgene Company (Addgene, USA). The construct pGFP-VPS34 was purchased from Miaoling Company (Miaoling, China). The authenticity of each construct was confirmed by DNA sequencing. The primers are listed in Table 1. Polyclonal antibody against CSFV Npro was kindly provided by Prof. Zhiyong Ma (Shanghai Veterinary Research Institute, Chinese Academy of Agricultural Sciences, China). Monoclonal antibodies against CSFV E2 protein were kindly provided by Prof. Qisheng Zheng (Jiangsu Academy of Agricultural Sciences, Institute of Veterinary Immunology & Engineering, China). All the other antibodies are listed in Table 2.

**TABLE 1:** The primers used in this study

Primer	Sequence (5′−3′)	Accession	Use
CSFV-F	CCTGAGGACCAAACACATGTTG	AF092448	RT-qPCR for the detection of CSFV
CSFV-R	TGGTGGAAGTTGGTTGTGTCTG		
JEV-F	AGAGCGGGGAAAAAGGTCAT	GQ918133	RT-qPCR for the detection of JEV
JEV-R	TTTCACGCTCTTTCTACAGT		
BVDV-F	GAGTACAGGGTAGTCGTCAGT	MG420995	RT-qPCR for the detection of BVDV
BVDV-R	CTCTGCAGCACCCTATCAGG		
PRV-F	GATGACCTCAACGGCGACCTC	MW805231	RT-qPCR for the detection of PRV
PRV-R	GCGAGAAGAGCTGCGAGTGG		
PEDV-F	GACGCGCTTCTCACTACTTC	OR734225	RT-qPCR for the detection of PEDV
PEDV-R	TGTACGCCAGTAGCAACCTT		
ACTIN-F	CTCCATCATGAAGTGCGACGT	AJ312193	RT-qPCR for the detection of ACTIN
ACTIN-R	GTGATCTCCTTCTGCATCCTGTC		
VPS34-F	GCACACAGAGCGAACAATACC	NM_001012956	RT-qPCR for the detection of VPS34
VPS34-R	GACTGCCTCTTCATCGGACA		
siVPS34-F	CUCCAAUGAAGCUGAAUAATT	NM_001012956	siRNA knockdown for VPS34
siVPS34-R	UUAUUCAGCUUCAUUGGAGTT		
siUVRAG-F	CUGAAUGAUGGCUAUUAUGTT	XM_021062526	siRNA knockdown for UVRAG
SiUVRAG-R	CAUAAUAGCCAUCAUUCAGTT		

**TABLE 2:** The antibodies used in this study

Antibody	Name	Supplier	Catalog no.
GAPDH	GAPDH monoclonal antibody	Proteintech	60004-1-Ig
GFP	GFP tag monoclonal antibody	Proteintech	66002-1-Ig
LC3	LC3 polyclonal antibody	Proteintech	14600-1-AP
P62	P62, SQSTM1 polyclonal antibody	Proteintech	18420-1-AP
Beclin 1	Beclin 1 polyclonal antibody	Proteintech	11306-1-AP
ATG5	ATG5 rabbit monoclonal antibody	ABclonal	A19677
VPS34	VPS34/PIK3C3 rabbit pAb	ABclonal	A12483
HA	Anti-HA antibody mouse MAb	Sigma-Aldrich	H6908
FLAG	Anti-FLAG M2 antibody	Sigma-Aldrich	F1804
β-actin	β-actin (C4) antibody	Santa Cruz	SC-47778
LAMP1	LAMP1 (D4O1S) mouse mAb	CST	15665

### Cell viability assay

Cells were seeded in a 96-well plate, and once the cell density reached 80%–90%, the compounds were diluted with 2% DMEM to different concentrations and maintained for 24 h. The cytotoxic effects of the compounds on cells were assessed using the Cell Counting Kit 8 (CCK-8, Absin Bioscience Inc., Shanghai, China). After incubation at 37°C for 1–4 h, the absorbance of the reagent was measured using a fluorescence microplate reader at 450 nm.

### Screening of glucose metabolism-targeted compounds

A glucose metabolism-targeted compound library was purchased from MedChem Express (MCE, USA) and stored as 10 mM stock solutions in DMSO at −80°C until use. Cells were treated separately with the compounds (1 µM compound or DMSO) for 24 h and then tested for cytotoxicity using a CCK-8 assay kit. During this screening, the compounds were removed if they showed observable cytotoxicity. For the next screening, cells were infected with CSFV (MOI = 1) and then treated separately with the compounds (1 µM) or DMSO. Fluorescence intensity was measured using ImageJ 7.0 software. The inhibition rate of each compound was normalized to the same volume of the DMSO-treated control group. Each assay was performed in triplicate. In this screening, compounds with an 80% inhibition rate of CSFV proliferation were selected.

### Binding and entry assay

Cells were pretreated with nontoxic concentrations of compounds at 37°C for 1 h and inoculated with CSFV (MOI = 10) at 4°C for 1 h to allow virus attachment without internalization. Subsequently, cells were washed three times with cold phosphate-buffered saline (PBS) before viral RNA was extracted and quantified by RT-qPCR (Binding). The culture medium was replaced with fresh serum-free DMEM, and cells were subsequently shifted to 37°C with 5% CO_2_ to allow virus internalization. After 1 h, cells were washed with citrate buffer solution (pH = 3) to remove noninternalized virions on the surfaces of cells and then washed three times with cold PBS before viral RNA was extracted and quantified by RT-qPCR (Entry).

### Time-of-addition assay

Cells were pretreated with the compound for 1 h before virus inoculation (1 h pre-treatment). Alternatively, the compound was added only during 1 h of infection, when most of the virus entry and fusion occurred (1 h entry), or after the 1 h of infection (after entry) and maintained for the remainder of the infection. Additionally, cells were pre-treated for 1 h, with the compound remaining throughout the 24 h of infection (throughout). After 24 h of incubation, cells were harvested for RT-qPCR and Western blotting.

### Reverse transcription real-time PCR

Total RNA was extracted from infected cells using TRIzol reagent (Invitrogen, CA, USA) and reverse-transcribed to cDNA using reverse transcription reagent (R222; Vazyme, Nanjing, China). Relative expression levels of target mRNA were determined by RT-qPCR using SYBR qPCR Master Mix (Q511; Vazyme, Nanjing, China). The expression levels of these genes were normalized to that of β-actin, and relative gene expression levels were calculated using the 2^−ΔΔCt^ method. The primers are listed in Table 1.

### Western blotting

Cells were harvested and lysed in radioimmunoprecipitation assay lysis buffer (R0020; Solarbio, Beijing, China) for 25 min at 4°C. Lysates were clarified by centrifugation at 12,000 × *g* for 10 min at 4°C, and the supernatant was then mixed with 5 × SDS loading buffer. Proteins in the lysates were separated by SDS-PAGE, transferred to nitrocellulose membranes, and probed with the indicated antibodies. β-actin or GAPDH was used as a loading control. To determine the indicated protein expression, grayscale analysis was performed with the corresponding amount of protein/β-actin or GAPDH by ImageJ 7.0 software.

### Plasmids and siRNA transfection

Cells grown to 60%–80% confluence in cell culture plates were transfected with specified plasmids using jetPRIME transfection reagent (Polyplus, France) as per the manufacturer’s protocol. For RNA interference, cells were transfected with siRNA using Lipofectamine RNAiMAX (Invitrogen, USA) according to the manufacturer’s instructions. All siRNA duplexes are listed in Table 1. They were designed and synthesized by GenePharma Co., Ltd (Shanghai, China) or Sangon (Shanghai, China). Cells were infected with CSFV (MOI = 1) and harvested for Western blotting at 24 hpi.

### Co-immunoprecipitation

Cells were transfected with indicated plasmids for 24 h and lysed in lysis buffer (50 mM Tris-HCl, pH 7.5, 150 mM NaCl, 1% Triton X-100, 1 mM EDTA, 1 mM PMSF) for 30 min at 4°C. The lysate was collected and centrifuged at 3,000 × *g* for 7 min at 4°C to obtain the supernatant (whole-cell lysate). A 100 µL aliquot of the supernatant was removed from all samples for later use. The remaining lysate was incubated with anti-GFP or HA antibody with rotation overnight, then the samples were incubated with agarose beads (Alpalifebio, China) and continuously rotated for 4–6 h at 4°C. The agarose beads were collected by centrifugation at 1,000 × *g* for 5 min at 4°C and then resuspended in 1 × SDS loading buffer for Western blotting.

### Confocal microscopy and IFA

Cells grown on coverslip dishes or 96-well plates were fixed with 4% paraformaldehyde for 30 min at room temperature and permeabilized with 0.1% Triton X-100 (Sigma, USA) for 15 min at room temperature. Cells were incubated with primary antibodies overnight at 4°C and then incubated with fluorescent-labeled secondary antibodies for 1 h at 37°C. The cell nuclei were stained with DAPI (Beyotime, China) for 10 min. Co-localization in 2D confocal images was assessed by calculating Pearson’s correlation coefficient using the proprietary analysis software integrated into the Nikon A1 system. For IFA, cells on 96-well plates were examined under a Zeiss LSM700 confocal microscope (Zeiss, Germany).

### Statistical analysis

All data were presented as means ± standard deviations (SD) as indicated. Student’s t test was used to compare data from treated and untreated groups. Asterisks in the figures indicate statistical significance (*p < 0.05; **p < 0.01; ***p < 0.001). All statistical analyses and calculations were conducted using GraphPad Prism 8.0 software.

## Data Availability

All relevant data are within the manuscript.
